# Mitochondria-Targeted Natural-Derived Compounds in Cellular Senescence: Mechanisms, Therapeutic Potential, and Future Directions

**DOI:** 10.3390/biology15151328

**Published:** 2026-08-06

**Authors:** Jirapat Namkaew, Pornparn Kongpracha, Thiranut Jaroonwitchawan

**Affiliations:** 1 Futuristic Science Research Center, School of Science, Walailak University, Thasala, Nakhon Si Thammarat 80160, Thailand; jirapat.na@mail.wu.ac.th; 2 Research Excellence Center for Innovation and Health Products (RECIHP), School of Allied Health Sciences, Walailak University, Thasala, Nakhon Si Thammarat 80160, Thailand; 3 School of Science, Walailak University, Thasala, Nakhon Si Thammarat 80160, Thailand; 4 Quantitative Biosciences Institute (QBI), University of California San Francisco, San Francisco, CA 94143, USA; jo.kongpracha@ucsf.edu; 5 Department of Cellular and Molecular Pharmacology, University of California San Francisco, San Francisco, CA 94143, USA; 6 Data Science and Biotechnology Institute, Gladstone Institutes, San Francisco, CA 94143, USA

**Keywords:** cellular senescence, mitochondrial dysfunction, natural bioactive compounds, mitochondria-targeted delivery

## Abstract

Cellular senescence is a root cause of aging and age-related disease. Senescent cells persist in tissues, secreting inflammatory factors that fuel inflammaging and immune decline. At the subcellular level, mitochondrial dysfunction has become recognized as a central driver of the senescent state: metabolism shifts toward glycolysis, mitophagy stalls while reactive oxygen species production escalates, mitochondrial dynamics tip toward hyperfusion or fragmentation, and damaged mitochondrial DNA leaks into the cytosol to activate the cyclic GMP-AMP synthase–stimulator of interferon genes pathway, amplifying the senescence-associated secretory phenotype. Conventional drugs have struggled to address these layered defects, steering interest toward natural bioactive compounds—polyphenols, flavonoids, saponins—that can simultaneously restore mitophagic flux, boost antioxidant defenses, rebalance fission–fusion, and intercept mitochondrial DNA-driven inflammation. However, the key issue is delivery: these molecules rarely reach mitochondria in meaningful concentrations in vivo due to their poor bioavailability, rapid metabolism, and off-target distribution. Platforms using triphenylphosphonium, mitochondria-penetrating peptides, or biomimetic shells have successfully funneled therapeutic payloads into mitochondria in several models of disease. We contend that the proposed systematic integration of these delivery systems with natural senotherapeutic compounds offers a promising direction for future research.

## Simple Summary

As we age, our bodies gather senescent cells that evade apoptosis and release detrimental signals, resulting in systemic inflammation and conditions such as dementia and heart failure. A significant contributor to their detrimental effects is the decline in mitochondrial function, which leads to the leakage of harmful substances and inflammatory reactions. Natural compounds derived from fruits, vegetables, and herbs have the potential to restore mitochondrial function and eliminate these senescent cells. Nevertheless, a significant obstacle is that these compounds seldom reach the mitochondria upon ingestion. This review explores micro-delivery systems that are designed to transport natural compounds directly to the mitochondria. Our findings indicate that both the natural repair compounds and delivery technologies exist, but surprisingly, no studies have yet combined them to target aging cells. We suggest the integration of these strategies to create innovative therapies that could decelerate the aging process and tackle various age-related diseases.

## 1. Introduction

Cellular senescence is now considered an active participant in the aging process rather than a passive bystander. Senescent cells build up in tissues and play a role in promoting age-related diseases through the senescence-associated secretory phenotype (SASP), which is a secretome that triggers chronic inflammation and immune system deterioration. Definitive evidence indicates that introducing senescent cells reduces lifespan, while eliminating them prolongs it. Despite the diverse nature of senescent cells, a common underlying factor at the subcellular level has been identified. Mitochondrial dysfunction, characterized by compromised mitophagy, increased reactive oxygen species (ROS) production, abnormal fission–fusion dynamics, and leakage of mitochondrial DNA that activates the immune activation pathway, is now acknowledged as both a cause and a consequence of cellular senescence. This shared mechanistic basis highlights mitochondria as a promising target for therapeutic interventions. Cellular senescence is progressively recognized as a promising target for pharmacological treatment in the context of aging and age-related diseases, establishing the essential scientific foundation that drives commercial interest and investment in this area. Natural bioactive compounds such as polyphenols, flavonoids, and saponins have the potential to enhance mitophagy, reduce oxidative stress, restore balance in mitochondrial dynamics, and inhibit inflammation triggered by mitochondrial DNA. The problem is not a shortage of active molecules, but it is delivery. These compounds rarely reach the mitochondrial matrix at effective concentrations in vivo due to poor bioavailability, rapid metabolism, and non-specific distribution. Emerging nanoparticle platforms that use triphenylphosphonium, mitochondria-penetrating peptides, or biomimetic carriers are beginning to address this bottleneck.

A comprehensive review has not been conducted to examine the specific ways in which natural bioactive substances impact mitochondrial functions such as mitophagy, dynamics, oxidative stress, and inflammation caused by mtDNA to combat cellular senescence. This review aims to address this gap by presenting the existing mechanistic information that connects mitochondrial irregularities with senescence. It also discusses the natural compounds that act on these mitochondrial processes and assesses the potential of nanoparticle-based delivery systems to effectively target these compounds to the desired subcellular locations. We propose that the strategic combination of delivery methods with natural anti-senescence treatments at multiple nodes, along with the development of sophisticated systems that limit mitochondrial activities to senescent cells, may represent a novel approach worthy of investigation in the field of geroprotective medicine.

## 2. Methodology

This narrative review was informed by a structured literature search of PubMed (the sole database used), covering 2021–2026. Additional references were obtained from the reference lists of retrieved articles. The literature search was performed using combinations of keywords including “cellular senescence”, “mitochondrial dysfunction”, “mitophagy”, “mitochondrial dynamics”, “mtDNA damage”, “cGAS-STING”, “SASP”, “inflammaging”, “natural bioactive com-pounds”, “polyphenols”, “flavonoids”, “senotherapeutics”, “mitochondria-targeted de-livery”, “triphenylphosphonium”, “mitochondria-penetrating peptides”, “nanoparticles”, and “biomimetic carriers”. Inclusion criteria comprised peer-reviewed original research and reviews addressing mitochondrial mechanisms in senescence, natural compounds with mitochondrial targets, or relevant delivery systems. Exclusion criteria were non-English articles, conference abstracts/editorials, and studies lacking mechanistic depth. Screening proceeded in two stages (title/abstract, then full-text), with disagreements resolved by consensus. To ensure methodological consistency, we prioritized SCOPUS Q1 journals, acknowledging this as a potential source of bias noted in our limitations. As this is a narrative review, PRISMA guidelines were not formally applied, though the structured approach ensures traceability.

## 3. Cellular Senescence in Aging and Age-Related Diseases

Cellular senescence has shifted from being seen as a passive hallmark of aging to being recognized as an active and causal driver of age-related diseases. In the past decade, significant evidence has clarified the mechanisms by which senescent cells impair the health of the organism. These cells are characterized by a stable arrest in proliferation, resistance to apoptotic clearance, persisting DNA damage signaling, telomere attrition, mitochondrial dysfunction, and most importantly, the development of SASP. The SASP is a complex array of secreted factors that are abundant in pro-inflammatory cytokines, chemokines, growth factors, and matrix-remodeling proteases. This secretome sustains a chronic, low-grade inflammatory state commonly referred to as “inflammaging” and contributes to the progressive decline of immune function known as immunosenescence [1,2,3]. Critically, senescent cells are not just passive accumulations resulting from aging; they actively contribute to the aging process. Several significant experiments have confirmed this causal link. For instance, Xu et al. showed that transplanting senescent mouse embryonic fibroblasts into middle-aged recipients shortened lifespan without elevating any single disease burden, implying that senescence accelerates systemic aging rather than being linked to a particular pathology [4]. In a parallel line of work, adoptive transfer of aged or DNA repair-deficient splenocytes into young mice was sufficient to elicit systemic senescence markers, enhance circulating SASP factors, and diminish lifespan. This is a striking example that an aged immune system alone can propel the aging of the organism [5]. Moreover, transplantation of aged bone marrow, or of senescent bone marrow macrophages, into young mice propagated senescence to distal metabolic tissues such as liver, muscle, and adipose tissue and triggered measurable metabolic and skeletal dysfunction [6]. These studies collectively indicate that senescence can be transmitted and has a systemic disruptive effect. The pathological impact of senescent cells is widespread throughout the body. Even though their growth is arrested, they remain metabolically active, and the ongoing release of SASP factors fosters tissue fibrosis, diminishes regenerative potential, and triggers secondary senescence in adjacent cells. In the central nervous system, senescent neurons, glial cells, and myeloid populations that reside in the brain are significant contributors to neuroinflammation and cognitive decline. A particularly instructive study found that genetic clearance of p16-positive senescent cells in the aged mouse brain rejuvenated the neuroimmune environment and preserved cognitive performance, restoring immune cell profiles to those observed in young females [7]. Together, these observations make a compelling case that targeting senescent cells is not simply a symptomatic intervention but a rational, mechanism-based strategy to counteract inflammaging, immunosenescence, and the multi-tissue functional erosion that characterizes aging.

## 4. Mitochondrial Dysfunction as a Central Hallmark of Senescence

### 4.1. Mitochondrial ROS and Impaired Mitophagy

**Figure 1:**
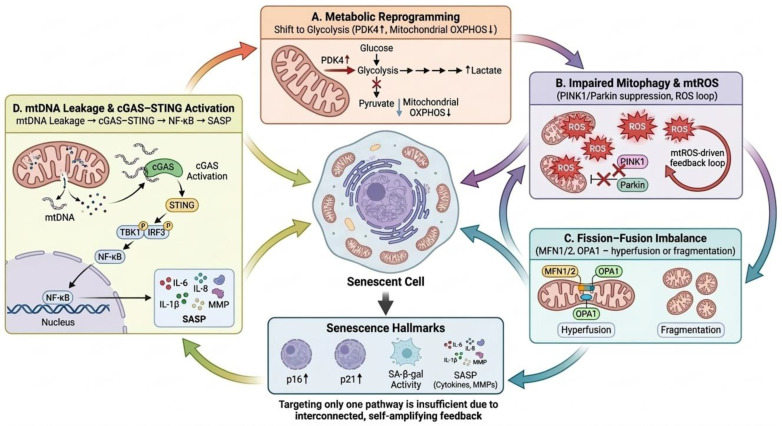
Mitochondrial dysfunction as a central hallmark of cellular senescence. Four interconnected mitochondrial mechanisms drive and sustain the senescent state. A feed-forward circuit of mitochondrial ROS and impaired mitophagy locks cells into stable senescence. Imbalanced mitochondrial dynamics—hyperfusion or excessive fragmentation—promotes organelle damage and SASP production. A senescent cell exhibits four self-amplifying mitochondrial and metabolic dysfunctions that feed back into each other: (**A**) Metabolic reprogramming (shift to glycolysis via PDK4); (**B**) Impaired mitophagy leading to mtROS buildup; (**C**) Mitochondrial fission-fusion imbalance; and (**D**) mtDNA leakage activating the cGAS-STING pathway through phosphorylation of TBK1 and IRF3, and NF-κB translocation to drive SASP secretion. The dots released from the damaged mitochondrion indicate stressed molecules. These pathways converge on classic senescence hallmarks (p16, p21, SA-β-gal, SASP). Solid arrows indicate activation or progression and thick arrows indicate major outcomes.

A fundamental question in senescence biology is how mitochondrial dysfunction arises and is perpetuated in senescent cells. A consistent observation across diverse models is that senescent cells exhibit increased mitochondrial mass or volume; however, this expanded mitochondrial pool is functionally compromised, exhibiting a reduced membrane potential and impaired respiration (Figure 1). Mitochondrial dysfunction has been documented in fetal airway smooth muscle cells under hyperoxia [8], in fibroblasts coping with proteostatic [9], in mesenchymal stem cells in high glucose [10], in vascular smooth muscle cells exposed to cholesterol [11], and in T lymphocytes pushed by oxidative stress [12]. The excess ROS then damages DNA, lipids, and proteins, feeding back to reinforce senescence pathways. Where does the circle start? A growing body of study suggests that impaired mitophagy is the critical link that connects mitochondrial ROS to the senescent phenotype. In senescent synovial mesenchymal cells under high glucose, fission is rampant, ROS are high, and mitophagic clearance is defective [10]. The same pattern appears in doxorubicin-induced cardiomyocyte senescence, where mitochondria are damaged, ROS are elevated, and simply restoring mitophagy rescues key features of senescence [13]. The regulatory layers that govern this axis are becoming increasingly intricate than was initially acknowledged. For instance, a deficiency in IDH2 results in the accumulation of miR-34b/c, which then inhibits PINK1, Parkin, LC3, and SIRT3, effectively ceasing mitophagy. This process subsequently promotes endothelial senescence through heightened mitochondrial ROS [14]. In a related pathway, SIRT4 levels diminish in senescent chondrocytes, leading to the inhibition of PINK1-mediated mitophagy, the accumulation of damaged mitochondria, an increase in ROS, and the onset of senescence; notably, the overexpression of PINK1 can reverse these abnormalities [15]. There is an additional nuance: basal mitophagy actually depends on physiological ROS levels, and the induction of senescence abruptly cuts mitophagic flux, trapping the cell in a state of persistent mitochondrial dysfunction [16]. The pathophysiological significance of this mechanism was sharply highlighted by the discovery of PD1^+^ CD4^+^ T cells in rheumatoid arthritis patients that display clear senescence markers; the adoptive transfer of these cells into an RA mouse model exacerbates senescence phenotypes through impaired mitophagy and the resulting overload of mtROS [17]. Taken together, the data describe a feed-forward circuit—mitochondrial ROS and defective mitophagy reinforce each other, locking the cell into a stable senescent state.

### 4.2. Mitochondrial Dynamics: The Balance Between Fusion and Fission

Mitochondrial dynamics, governed by the opposing processes of fusion and fission, are tightly regulated to maintain cellular homeostasis (Figure 1). Disruption of this balance, whether favouring excessive fusion or excessive fission, can drive cellular senescence or cell death. In the majority of senescent cells, mitochondrial dynamics are skewed towards hyperfusion, resulting in the accumulation of damaged mitochondria, impaired mitophagy, and elevated mitochondrial ROS, thereby establishing a vicious cycle that amplifies the senescent phenotype; extreme hyperfusion may ultimately trigger cell death. Mitochondrial fusion is primarily mediated by the dynamin-related GTPases mitofusin 1 (MFN1) and mitofusin 2 (MFN2), located on the outer mitochondrial membrane. In chemotherapy-induced senescent melanoma cells, MFN1/2 levels rise and mitochondrial fusion increases; this hyperfused state is required for the full expression of the SASP. Accordingly, MFN1 knockdown reduces IL-6 and other immunomodulatory SASP factors and enhances immune-mediated clearance of tumours following therapy [18]. In liver cancer, disruption of the HELLS–MIEF1 axis, which normally maintains fission homeostasis, leads to mitochondrial hyperfusion, energy deprivation, and a senescence-like state characterised by low ROS and restrained tumour growth [19]. Recent research has revealed additional evidence that the inhibition of glutaminase 1 (GLS1) in senescent renal tubular cells leads to MFN1-dependent hyperfusion. This process results in an increased generation of reactive oxygen species (ROS) and the activation of the mitochondrial permeability transition pore (mPTP), which selectively removes senescent cells (senolysis) and contributes to the acceleration of kidney aging in mice [20]. Thus, sustained mitochondrial fusion in senescent or cancer cells can either support a pro-tumorigenic SASP or promote senescent cell death, positioning the fusion machinery as a nuanced but powerful senotherapeutic target.

Mitochondrial fission is mainly facilitated by dynamin-related protein 1 (DRP1), a GTPase that is recruited to the mitochondrial surface by adaptor proteins such as FIS1, MFF, and MID49/51, playing a critical role in mitophagy and mitochondrial quality control. In senescence, fission is frequently dysregulated, either suppressed or excessively activated, with distinct consequences for SASP and cell fate. In rheumatoid arthritis patients, PD1^+^ senescent CD4^+^ T cells exhibit a reduction in DRP1 levels, an increase in OPA1, and elongated, hyperfused mitochondria. This is associated with the accumulation of mitochondrial ROS, impaired mitophagy, and a significant SASP. Mechanistically, the PD1–HIF1α signaling pathway inhibits the transcription of DRP1, thereby promoting this altered cellular state [17]. Conversely, in 5-FU-induced senescent colorectal cancer cells, treatment with piceatannol shifts mitochondria towards a fragmented morphology by downregulating MFN1/2 and strongly upregulating the DRP1 adaptor MFF, leading to mitochondrial depolarisation and apoptosis-inducing factor (AIF)-dependent apoptosis that selectively eliminates senescent cells over non-senescent counterparts [21]. However, insufficient fusion can also promote senescence. In COPD lung tissue, MFN2 and OPA1 are downregulated, mitochondria are smaller and more numerous (fragmented), and senescence and SASP markers (p16, γH2A.X, IL-6, IL-1β, CXCL1/8) are elevated [22]. Similarly, in human mesenchymal stem cells, OPA1 loss causes mitochondrial fragmentation, dysfunction, and premature senescence, whereas restoring OPA1 rescues mitochondrial structure and delays senescence [23]. These findings illustrate that while restoring appropriate fission can dampen senescence, pushing already damaged mitochondria towards further fragmentation can be exploited to selectively eliminate senescent cells, highlighting the dual and context-dependent roles of mitochondrial dynamics in senescence regulation.

### 4.3. MtDNA Damage and Innate Immune Activation

Mitochondrial damage initiates a self-perpetuating vicious cycle, wherein the overproduction of ROS leads to mtDNA damage and mutations. This, in turn, further undermines mitochondrial function and ultimately results in genomic DNA damage, which strengthens the senescent phenotype through various mechanisms. As age progresses, mtDNA mutations accumulate, and when they achieve high levels of heteroplasmy, they are causally associated with relevant mitochondrial dysfunction and aging-like characteristics. The analysis of single-cell mtDNA sequencing in aged human blood cells has revealed that more than 50% of B cells and monocytes carry at least one mutation with a variant allele frequency greater than 20%. Numerous mutations identified are expected to be highly pathogenic [24]. Pathogenic mtDNA point mutations compromise the respiratory chain, resulting in diminished membrane potential and ATP synthesis, while concurrently elevating mtROS production. Notably, the interplay between excess mtROS and mtDNA mutations is both reciprocal and self-reinforcing: ROS facilitates mtDNA damage, and the respiratory chain subunits encoded by the damaged mtDNA produce additional ROS. A diabetes-related mitochondrial mutation serves as an example of this cycle, causing mitochondrial dysfunction through reduced complex I activity, a decrease in ATP and membrane potential, a lowered NAD^+^/NADH ratio, and heightened ROS production [25]. Beyond its local energetic consequences, damaged mtDNA acts as a potent pro-inflammatory signal when released into the cytosol. Under conditions of mitochondrial stress, mtDNA escapes into the cytoplasm where it functions as a danger-associated molecular pattern (DAMP), alerting innate immune DNA sensors to the presence of cellular damage. Three principal sensing pathwaysc—GAS-STING, TLR9, and the NLRP3 inflammasome—recognise cytosolic mtDNA and translate mitochondrial damage into inflammatory responses that drive and propagate senescence.

The cGAS-STING pathway serves as a central DNA-sensing mechanism linking mitochondrial damage to the SASP (Figure 1). Cytosolic mtDNA binds and activates cyclic GMP-AMP synthase (cGAS), which triggers STING-dependent signalling, culminating in the production of type I interferons and pro-inflammatory cytokines such as IL-6, TNF-α, and IL-1β, thereby inducing senescence-like phenotypes. Genetic inhibition of STING reduces SASP expression and senescence burden in aged mice, and STING activation has been specifically localised to microglia in the hippocampus of aged mouse brain, where it drives type I IFN and pro-inflammatory gene expression [26]. Blocking VDAC oligomerisation on the outer mitochondrial membrane can reduce mtDNA release and dampen both cGAS-STING activation and the SASP, confirming the mechanistic link between mtDNA efflux and inflammatory senescence [27]. The role of this pathway is compellingly highlighted by the induction of T-cell senescence through a *Salmonella* genotoxin, which is facilitated by the activation of cGAS-STING in response to the release of mtDNA and the subsequent generation of SASP, thus transmitting senescence to neighboring cells [28]. The importance of this axis extends beyond the inherent damage to mtDNA: ATM-deficient mice, which exhibit a defect in nuclear DNA repair, reveal an accelerated senescence of fibroblasts. This occurrence is propelled by continuous DNA damage signaling via the cGAS-STING pathway, highlighting the connection between nuclear and mitochondrial DNA damage within this framework [29].

The NLRP3 inflammasome provides an additional layer of innate immune activation in response to mitochondrial stress. Oxidised mtDNA is released from stressed mitochondria via mPTP opening and VDAC-dependent efflux, after which it directly binds NLRP3, promoting inflammasome assembly. This process engages cGAS-STING pathway activation, creating a signalling network that amplifies the inflammatory response to mitochondrial damage [30]. TLR9, traditionally associated with innate immune defence, has also been implicated in senescence regulation. In breast cancer cells, TLR9 expression is reduced, and its restoration decreases proliferation while increasing senescence markers, including SA-β-gal staining and SASP components such as IL-6, IL-8, CXCL1, and GM-CSF, suggesting a functional role for TLR9 in the senescence programme [31]. Therefore, mitochondrial damage acts as a powerful trigger for cellular senescence by releasing mtDNA into the cytosol, where it engages a suite of innate immune sensors—notably cGAS-STING, NLRP3, and TLR9 that orchestrate the chronic inflammatory SASP. This immunostimulatory cascade reinforces the senescent state and propagates senescence to surrounding tissues, positioning the mtDNA-innate immune axis as both a fundamental driver of inflammaging and a promising therapeutic target.

### 4.4. Metabolic Reprogramming in Senescence

Mitochondrial dysfunction is a hallmark of cellular senescence that drives profound alterations in cellular metabolism, ultimately contributing to age-related diseases and tissue damage. Senescent cells, despite being arrested in the cell cycle, remain metabolically active and undergo extensive metabolic rewiring to sustain their survival and support energy-demanding features such as the SASP. This reprogramming is typically characterised by a shift from oxidative phosphorylation (OXPHOS) towards increased glycolysis (Figure 1), glutaminolysis, altered fatty acid oxidation (FAO), and enhanced branched-chain amino acid degradation. A prominent feature of this metabolic shift is the “Warburg-like” upregulation of glycolysis. Senescent stromal cells exhibit an upregulation of pyruvate dehydrogenase kinase 4 (PDK4), which leads to increased lactate production that, in turn, promotes the proliferation of neighboring tumor cells through NOX1-dependent ROS generation; pharmacological inhibition of PDK4 has been shown to decrease tumor progression in vivo, thereby affirming the functional relevance of this metabolic pathway [32]. Despite a decrease in OXPHOS activity, the mitochondrial proteome of senescent cells reveals an enrichment in pathways related to branched-chain amino acid degradation, suggesting a compensatory reliance on alternative energy sources amidst the accumulation of mitochondrial dysfunction [33]. Furthermore, the responses to DNA damage can trigger direct modifications in mitochondria that facilitate senescence. The phosphorylation of the mitochondrial membrane protein BNIP3 results in the remodeling of cristae, which boosts fatty acid oxidation, thereby supplying the acetyl-CoA necessary for the sustained expression of p16 [34]. In addition, senescent stem cells exhibit an increased dependence on glutamine catabolism, leading to elevated urea production that further compromises mitochondrial integrity. Crucially, the inhibition of glutaminase (GLS1) activity has been demonstrated to improve mitochondrial function and reduce various aging-related traits, thus restoring a more favorable metabolic environment [35]. These metabolic modifications, which encompass glycolysis, lipid oxidation, amino acid catabolism, and glutamine utilization, not only preserve the senescent phenotype but also present promising therapeutic targets for the enhancement of mitochondrial health and the alleviation of senescence.

## 5. Current Therapeutic Limitations and Natural Products as Alternatives

Restoring or preserving mitochondrial function holds the promise of alleviating various hallmarks of cellular senescence across diverse cell types and experimental models. At present, interventions focused on mitochondria can be classified into three primary categories: enhancing mitochondrial metabolism and biogenesis, improving mitochondrial quality control, and reducing oxidative stress. For example, the pharmacological stabilization of hypoxia-inducible factor 1α (HIF-1α) via dimethyloxalylglycine (DMOG) has demonstrated the ability to restore mitochondrial morphology, membrane potential, and respiratory efficiency in senescent mesenchymal stem cells, while also facilitating mitophagy and lowering significant senescence markers such as p53 and p21 [36]. Mitochondria-targeted antioxidants, including MitoTEMPO and MitoQ, effectively scavenge mitochondrial ROS, limit oxidative DNA damage, and preserve organelle function, thereby delaying senescence onset [37,38]. Similarly, strategies aimed at restoring autophagic flux, such as AKT inhibition with GDC0068, enhance lysosomal function and promote clearance of damaged mitochondria, leading to improved membrane potential and reduced senescence burden [39].

Although there are promising pharmacological proofs of concept, synthetic agents that target mitochondria often encounter limitations such as off-target effects, chronic toxicity issues, and narrow mechanisms of action that only address singular aspects of mitochondrial dysfunction. Natural products, including polyphenols, flavonoids, terpenoids, alkaloids, and carotenoids, offer a compelling alternative. These compounds have been evolutionarily optimized for biological interaction, often demonstrating multi-target capabilities that can simultaneously modulate oxidative stress, mitophagy, mitochondrial biogenesis, and metabolic flux, and generally possess favorable safety profiles established through long-term dietary exposure. Nonetheless, a dedicated review that specifically examines their role in mitigating cellular senescence through mitochondrial pathways is currently lacking. This gap is particularly significant given that the therapeutic potential of natural products is often severely constrained by poor solubility, low bioavailability, and insufficient mitochondrial accumulation issues that could, in principle, be addressed by the advanced targeting strategies discussed in this review.

## 6. Natural Bioactive Compounds Counteract Senescence Through Mitochondrial Mechanisms

### 6.1. Targeting Impaired Mitophagy

The dysfunction of mitophagy is a primary factor that leads to the accumulation of dysfunctional mitochondria and the establishment of the senescent phenotype. Thus, restoring mitophagic flux is a rational therapeutic approach for reversing cellular senescence. An expanding body of preclinical evidence shows that various natural bioactive molecules can enhance mitochondrial quality control through mitophagy, effectively reducing or reversing senescence phenotypes in diverse cell types. These compounds converge on a series of evolutionarily conserved regulatory pathways, particularly PINK1/Parkin, BNIP3, SIRT1, and AMPK.

The PINK1/Parkin axis is well-established as a central component in the failure of mitophagy associated with senescence. In primary human fibroblasts that have been modified with a mitophagy reporter, the baseline activity of PINK1/Parkin-p62-mediated mitophagy is robust; however, this activity is significantly suppressed within 11 days following senescence induced by irradiation [16]. The restoration of mitophagic flux through supplementation with NAD^+^ precursors occurs solely via a PINK1/Parkin-dependent mechanism, which provides a direct mechanistic association between the restoration of mitophagy and the reversal of senescence [16]. The in vivo relevance of this pathway is further reinforced by investigations into herbicide-induced kidney senescence in mice, where melatonin effectively rescues the senescent phenotype in wild-type mice, but Parkin-deficient mice show no response, confirming that a functional PINK1/Parkin axis is crucial for the protective effect [40].

**Figure 2:**
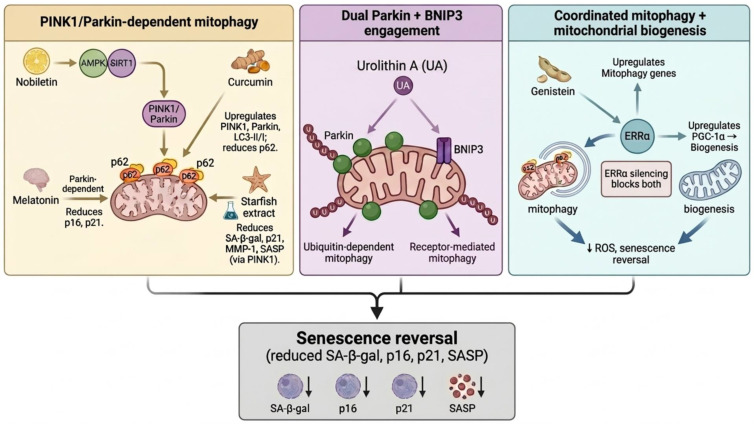
Natural bioactive compounds counteract senescence through mitochondrial mechanisms. The figure depicts three mechanistically distinct strategies by which natural compounds restore mitochondrial health in senescent cells. (**Left Panel**) PINK1/Parkin-dependent mitophagy. Four structurally diverse compounds—nobiletin, melatonin, curcumin, and a starfish-derived marine extract—converge on the PINK1/Parkin axis to enhance mitophagic clearance, identifying this pathway as a broadly accessible pharmacological node. (**Middle Panel**) Dual Parkin and BNIP3 engagement. Urolithin A, a gut-microbiome-derived ellagitannin metabolite, activates both the ubiquitin-dependent (Parkin) and receptor-mediated (BNIP3) arms of mitophagy, achieving broader mitochondrial quality control than PINK1/Parkin-restricted compounds. (**Right Panel**) Coordinated mitophagy and mitochondrial biogenesis. Genistein, a soy isoflavone, acts through ERRα to simultaneously clear damaged mitochondria via mitophagy and generate new organelles via biogenesis, representing a comprehensive clearance-and-renewal strategy. Solid arrows indicate activation or progression.

Four biosynthetically diverse natural compounds including nobiletin, melatonin, curcumin, and a starfish extract collectively target the PINK1/Parkin mitophagy pathway through their diverse biosynthetic properties (Figure 2). The finding that chemically distinct compounds exert their anti-aging effects via a shared mechanism indicates that PINK1/Parkin could function as a broadly available pharmacological target; however, this hypothesis requires further validation in additional models and, ultimately, in human studies. Nobiletin, a citrus-derived polymethoxyflavonoid, protects against D-galactose-induced granulosa cell aging in the chicken ovary [41]. Mechanistically, it acts by activating the AMPK/SIRT1 signalling cassette upstream of PINK1/Parkin-mediated mitophagy; pharmacological blockade of either AMPK or SIRT1 entirely abolishes its anti-senescence efficacy [41]. This stringent upstream reliance suggests that nobiletin does not bypass the canonical signaling hierarchy but instead interacts with it at an elevated regulatory level. In addition, melatonin, as noted, prevents atrazine-induced renal tubular senescence, and its capacity to reduce p16 and p21 expression is strictly Parkin-dependent [40]. Curcumin expands this mechanistic pathway to a different cell type. Curcumin, delivered via liposomal carriers to D-galactose-treated senescent bone marrow-derived mesenchymal stem cells, upregulates PINK1, Parkin, and the autophagosome marker LC3-II/I, while concurrently reducing the adaptor protein p62; the protective effect is largely blocked by pharmacological inhibitors of mitophagy [42]. Finally, a bioactive extract from starfish reduces SA-β-gal, p21, MMP-1, and the SASP cytokines IL-6 and IL-8 in senescent human dermal fibroblasts, and shRNA-mediated knockdown of PINK1 abrogates these benefits [43]. That such chemically disparate compounds—a citrus flavonoid, an endogenous melatonin, a curcuminoid, and a marine natural product all rely on PINK1/Parkin activity strongly suggests that this pathway constitutes a broadly accessible and pharmacologically tractable node for attenuating cellular senescence.

A second group of compounds functions through mechanistically different pathways that either extend beyond PINK1/Parkin or merge mitophagy with mitochondrial biogenesis (Figure 2). Urolithin A (UA), a metabolite derived from ellagitannins by the gut microbiome, is a significant example. In the context of H_2_O_2_-induced auditory cell senescence, UA lowers levels of p53, p21, and SA-β-gal while simultaneously restoring mitochondrial ATP levels, mtDNA copy number, and membrane potential. In contrast to the previously discussed compounds, UA requires both Parkin and the outer mitochondrial membrane receptor BNIP3 for its anti-senescence effects; the silencing of either protein via siRNA disrupts its effect [44]. This dual reliance indicates that UA triggers both the ubiquitin-dependent and receptor-mediated aspects of mitophagy, leading to a more extensive activation of the mitochondrial quality control machinery than compounds that are solely focused on PINK1/Parkin.

Genistein, a soy-based isoflavone, utilizes a more holistic approach (Figure 2). In senescent bone marrow-derived mesenchymal stem cells derived from ovariectomized rats, a model that replicates both endocrine aging and mitochondrial decline, genistein alleviates ROS accumulation, mitochondrial dysfunction, and premature senescence through an ERRα-dependent mechanism. Notably, ERRα signaling simultaneously enhances the mitophagic clearance of impaired mitochondria and stimulates mitochondrial biogenesis to replenish the organelle reservoir [45]. The inhibition of ERRα disrupts both processes and negates the anti-senescence phenotype, indicating that genistein harmonizes the processes of removal and renewal. This integrated clearance-regeneration strategy represents a distinctly different homeostatic intervention not found with PINK1/Parkin-limited compounds.

In spite of their structural diversity, the six compounds can be grouped into three mechanistically defined strategies for mitophagy-based anti-senescence: PINK1/Parkin-dependent mitophagy alone, a combination of Parkin and BNIP3 engagement, and a dual program that encompasses both mitophagy and mitochondrial biogenesis. Across all categories, it has been demonstrated that functional mitophagy is necessary rather than merely associated with the anti-senescence effect. However, it is important to acknowledge that most of the existing evidence is derived from chemically induced senescence models or stressed primary cells maintained in culture, along with relatively short-term in vivo interventions. Whether these mechanisms function with comparable efficacy in naturally aged tissues, where senescent cells accumulate over the years in a complex, chronically inflamed microenvironment, remains an open and urgent question.

### 6.2. Protecting Mitochondria via Antioxidative Mechanisms

**Figure 3:**
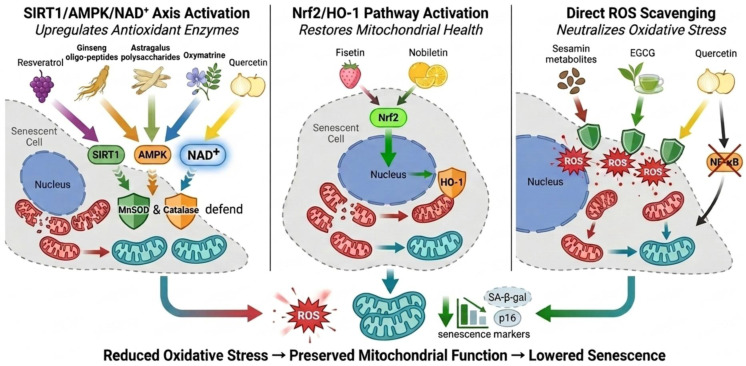
Natural compounds protect mitochondria and attenuate senescence through three antioxidative strategies. (**Left panel**) Five compounds: resveratrol, ginseng oligopeptides, astragalus polysaccharides, oxymatrine, and quercetin activate the SIRT1/AMPK/NAD^+^ axis, upregulating antioxidant enzymes (MnSOD, Catalase) and preserving mitochondrial function. (**Middle panel**) Fisetin and nobiletin restore mitochondrial health via the Nrf2/HO-1 pathway. (**Right panel**) Sesamin metabolites, EGCG, and quercetin act as direct ROS scavengers, with quercetin also suppressing NF-κB. All three strategies converge on reduced oxidative stress, preserved mitochondrial function, and lowered senescence markers. Solid arrows indicate activation or progression; thick arrows indicate major outcomes; dashed arrows indicate translocation; x indicates inhibition or suppression.

A diverse array of plant- and food-derived compounds defend mitochondria, act as antioxidants, and reduce markers of cellular aging in cell and animal models. As illustrated in Figure 3, nine compounds have been identified that protect against cellular senescence through three functional strategies: activation of the SIRT1/AMPK/NAD^+^ axis, Nrf2/HO-1-mediated mitochondrial restoration, and direct ROS scavenging. All three strategies converge on reduced oxidative stress, preserved mitochondrial function, and eventually lowered senescence phenotypes.

#### 6.2.1. SIRT1/AMPK/NAD^+^ Axis Activation

Resveratrol, ginseng oligopeptides, astragalus polysaccharides, oxymatrine, and quercetin, which function through SIRT1 in specific contexts, engage with upstream pathways associated with longevity. Resveratrol is the most extensively studied in this group, stimulating the signals ofSIRT1/FOXO3 and SIRT1-Nrf2-FOXO3, leading to an increase in antioxidant enzymes such as catalase, SOD, and GSH-Px. Concurrently, resveratrol diminishes oxidative stress, inflammation, and ferroptosis in skin and ovarian models. Furthermore, it hinders MAPK signaling and decreases the levels of COX-2 and MMP expression [46,47,48].

Functioning through a parallel but distinct route, ginseng oligopeptides enhance mitochondrial function and biogenesis by acting on the NAD^+^/SIRT1/PGC-1α pathway, thereby reducing levels of p16 and p21 while boosting telomerase activity [49]. Astragalus polysaccharides, by contrast, restore mitochondrial membrane potential and enhance total antioxidant capacity through SIRT1-p53 signalling in aged aortic endothelium [50]. Oxymatrine adds yet another dimension: it reduces ROS, reactivates AMPK, activates the Nrf2-SIRT3 pathway, and improves mitochondrial function in models of brain and cardiac senescence [51,52]. Quercetin, the most multifaceted among the five compounds, can reduce ROS, SA-β-gal activity, p53/p21 expression, and inflammatory cytokines by interacting with SIRT1. However, the mechanism of action varies based on the specific cellular environment [53,54,55,56].

The commonality among these five compounds lies not only in their interaction with SIRT1 but also in their ability to enhance the expression of antioxidant enzymes while effectively maintaining various mitochondrial aspects such as membrane potential, biogenesis, and respiratory function. This persistent correlation indicates that focusing on the SIRT1/AMPK/NAD^+^ pathway could serve as a potentially effective preclinical strategy to mitigate oxidative stress and improve mitochondrial function in aging cells; however, further validation in naturally aged tissues and human models is necessary.

#### 6.2.2. Nrf2/HO-1-Mediated Mitochondrial Restoration

While the SIRT1/AMPK/NAD^+^ pathway plays a significant role in mitochondrial protection associated with longevity, another pathway, Nrf2/HO-1 signaling, operates in parallel. Fisetin and nobiletin elevate the activity of antioxidant enzymes by activating the Nrf2/HO-1 pathway, rejuvenate mitochondrial performance, and decrease levels of p53 and p21, ultimately easing the process of follicle aging [57]. Their ability to combat aging is intricately linked to enhancing mitochondrial well-being and diminishing oxidative harm. The question of whether the combination of SIRT1 activators with Nrf2/HO-1 inducers results in additional benefits or enhanced effects is currently unresolved but holds significant practical implications.

#### 6.2.3. Direct ROS Scavenging

Not all compounds that protect mitochondria depend on signaling cascades situated upstream. Another category, which includes sesamin metabolites, EGCG, and quercetin, operates more directly by scavenging mtROS without always involving the genetic programs that characterize the SIRT1 or Nrf2/HO-1 pathways. Sesamin metabolites lower mitochondria-derived ROS, improve the ROS/ATP ratio, and reduce SASP factors and DNA damage [58]. The primary catechin found in green tea, EGCG, has a wide-ranging effect. It inhibits SA-β-gal activity, reverses the age-related increase in p21, p16, and p15, and notably reduces IL-6 and IL-8 release, while also balancing the pro-inflammatory bias of monocytes when exposed to secretions from senescent endothelial cells. [59]. Additionally, quercetin, when functioning through NF-κB suppression, can reduce inflammatory cytokines and oxidative stress markers [56].

The SIRT1/AMPK/NAD^+^ axis is the most frequently engaged upstream regulator and consistently couples antioxidant enzyme upregulation with mitochondrial protection. The Nrf2/HO-1 pathway serves as a parallel node while direct ROS scavengers operate independently of these signaling axes. A critical translational gap persists: the vast majority of these studies employ chemically induced or oxidative stress-induced senescence models, and validation in naturally aged tissues or human samples remains lacking.

### 6.3. Restoring Mitochondrial Fission–Fusion Dynamics

Mitochondrial morphology is dynamically shaped by biological responses to nutritional cues, metabolic changes, infection, and the aging process. The balance between mitochondrial fission and fusion is therefore tightly linked to cellular senescence, and disturbed dynamics are now recognized as a part of the core senescence mechanism [60,61]. In most senescent cells, there is a tendency towards increased fusion of mitochondria. Instead of undergoing fragmentation and removal as they should be, mitochondria in these cells become elongated, enlarged, and functionally impaired. Due to the fact that fission is necessary for mitophagy, fused mitochondria avoid quality control mechanisms, leading to the accumulation of oxidative damage and the release of ROS into the cytoplasm. This sets off a harmful cycle wherein ROS inhibits mitophagy, and dysfunctional mitophagy allows ROS-producing mitochondria to persist. It is important to note, however, that excessive fission, on the other end of the spectrum, can also contribute to dysfunction and senescence in specific situations, highlighting that the issue lies not in fusion or fission themselves, but in the disruption of a responsive, balanced state.

This detailed understanding positions mitochondrial dynamics as an appealing yet insufficiently explored target for interventions to combat cellular senescence. The rationale is clear: if disrupted dynamics contribute to cell senescence, then restoring a balance between fission and fusion should theoretically aid in reversing this process.

**Figure 4:**
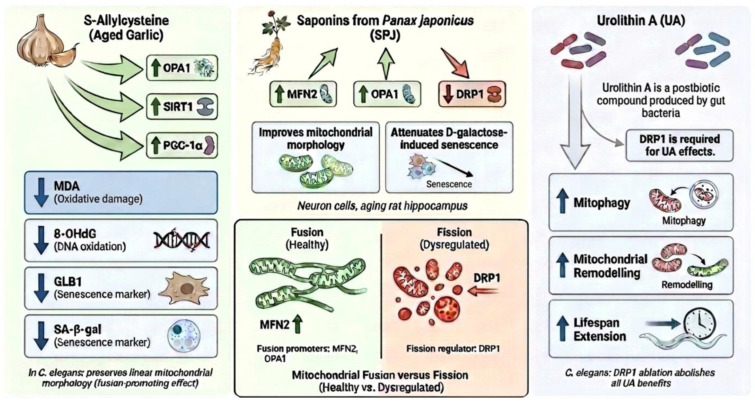
Natural compounds restore mitochondrial fission–fusion dynamics to attenuate senescence. (**Left panel**) S-allylcysteine (aged garlic) upregulates OPA1, SIRT1, and PGC-1α, preserving mitochondrial morphology and reducing oxidative damage and senescence markers in naturally aged mice and *Caenorhabditis elegans* (*C. elegans*). (**Middle panel**) *Panax japonicus* saponins upregulate MFN2 and OPA1 while downregulating DRP1, improving mitochondrial morphology and attenuating senescence in neuronal cells and aging rat hippocampus. (**Right panel**) Urolithin A functionally engages DRP1 without altering its expression, and DRP1 ablation abolishes its mitophagy-promoting and lifespan-extending effects in *C. elegans*. Solid arrows indicate activation or progression; thick arrows indicate major outcomes.

Emerging experimental evidence demonstrates that natural bioactive compounds can modulate mitochondrial dynamics to counteract senescence. The mitochondrial fusion proteins MFN2 (outer membrane) and OPA1 (inner membrane) are central to mitochondrial quality control and have been identified as targets of several natural products (Figure 4). A compelling example comes from aged garlic. S-allylcysteine, a bioactive compound derived from aged garlic, increased hepatic OPA1 mRNA expression alongside the mitochondrial regulators SIRT1 and PGC-1α in naturally aged mice [62]. These molecular changes were accompanied by reduced oxidative damage markers (MDA, 8-OHdG) and decreased senescence markers including galactosidase beta 1 (GLB1) and SA-β-gal. In *Caenorhabditis elegans*, S-allylcysteine preserved linear mitochondrial morphology, indicative of healthier fusion dynamics [62].

Likewise, saponins derived from *Panax japonicus* (SPJ) increased the expression of MFN2 and OPA1, while reducing the levels of the fission protein DRP1 in the hippocampus of elderly rats. This led to enhancements in mitochondrial structure and a reduction in D-galactose-induced cellular aging in SH-SY5Y neuroblastoma cells [63]. While promoting fusion has received most of the attention so far, the aspect of fission is equally crucial but has not been extensively studied. The direct regulation of DRP1 levels by natural substances has limited documentation. Nevertheless, research indicates that urolithin A (UA) can influence the activity and role of DRP1 without changing its levels or location within cells. UA relies on DRP1 to produce its effects: blocking DRP1 through drugs or genetic methods eliminates UA-triggered processes such as mitophagy, restructuring of mitochondria, and lifespan extension in *C. elegans* [64].

Collectively, these results indicate that natural bioactive substances are capable of restoring the equilibrium of mitochondrial dynamics. This can be achieved by enhancing the expression of fusion proteins (MFN2, OPA1) or by actively involving the fission machinery (DRP1) to maintain mitochondrial health and reduce the effects of aging. Despite the current findings, the number of studies that focus on the direct examination of fission–fusion modulation through natural products in senescence models is quite limited, highlighting the need for further exploration to determine if this mechanistic pathway can be effectively harnessed for anti-senescence applications.

### 6.4. Blunting mtDNA-Driven Inflammatory Signalling

**Figure 5:**
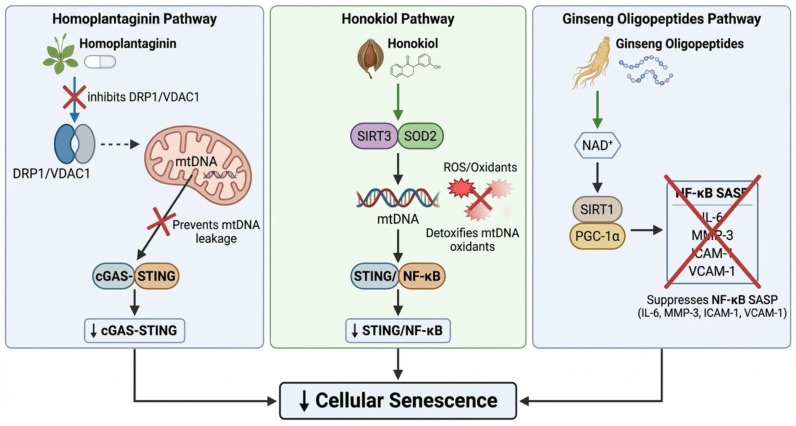
Natural bioactive compounds blunt mtDNA-driven inflammatory signalling at three intervention points. (Node 1) Homoplantaginin inhibits DRP1-dependent fission and VDAC1 oligomerisation, preventing mtDNA leakage into the cytosol and suppressing cGAS-STING activation. (Node 2) Honokiol activates SIRT3, enhancing SOD2-mediated detoxification of mtDNA-damaging oxidants within the mitochondrial matrix, thereby reducing cGAS-STING-NF-κB signalling. (Node 3) Ginseng oligopeptides act downstream via the NAD^+^/SIRT1/PGC-1α axis to suppress NF-κB-driven SASP factors. Together, these compounds intercept the mtDNA-cGAS-STING-NF-κB cascade at multiple levels—upstream at mtDNA release, midstream at antioxidant defence, and downstream at SASP execution. Solid arrows indicate activation or progression; thick arrows indicate major outcomes; dashed arrows indicate redistribution of a molecule from one cellular compartment to another; x indicates inhibition or suppression.

When mitochondrial membranes are breached, mitochondrial DNA is released into the cytosol, activating a strong innate immune reaction via the cGAS-STING-NF-κB pathway, thus stimulating the SASP and enhancing cellular senescence (Figure 5). This series of events offers various opportunities for intervention. Specifically, natural bioactive compounds act by blocking the cGAS-STING-NF-κB axis. The initial and most direct intervention involves preventing the leakage of mtDNA. For instance, homoplantaginin, a flavonoid obtained from *Salvia plebeia*, disrupts this process at its inception by impeding DRP1-mediated mitochondrial division and VDAC1 aggregation, consequently diminishing the release of mitochondrial DNA into the cytoplasm [65]. In mice with diabetes and human umbilical vein endothelial cells (HUVECs) exposed to high glucose levels, the mentioned intervention inhibited the cGAS-STING signaling pathway and subsequent inflammatory agents, leading to a decrease in SA-β-gal activity and a weakened SASP [65].

A second strategy is to fortify the mitochondrial matrix against oxidative damage. Honokiol, a polyphenol derived from *Magnolia officinalis*, exerts its effects further along the pathway but with similar therapeutic outcomes. In an experimental model of silicosis, honokiol activated the mitochondrial deacetylase SIRT3, enhancing the antioxidant capabilities of SOD2 and reducing oxidative damage to mtDNA directly within the mitochondrial matrix. This decline in impaired mtDNA levels resulted in the downregulation of cGAS and the inhibition of STING/NF-κB activation, ultimately reducing senescence in alveolar type II cells and pulmonary fibrosis [66].

A third, broader strategy operates further downstream. Ginseng oligopeptides enhance the strategies aimed at combating stress by postponing the aging of fibroblasts induced by oxidative stress through a comprehensive homeostatic programme. They protect the integrity of the genetic material, improve the performance of mitochondria through the NAD^+^/SIRT1/PGC-1α pathway, and inhibit the secretion of SASP factors driven by NF-κB, such as IL-6, MMP-3, ICAM-1, and VCAM [49]. Together, these three instances illustrate that natural bioactive substances have the ability to impact the mtDNA-cGAS-STING-NF-κB pathway at various stages, initially during mtDNA discharge, subsequently through antioxidant protection, and finally at the point of SASP activation, presenting a consistent, multi-faceted treatment strategy for combating senescence driven by mitochondria. The natural compounds discussed in this section, along with their sources, target pathways, experimental models, and principal findings, are summarized in Table 1.

### 6.5. Critical Appraisal of Natural Compounds: Evidence Strength and Knowledge Gaps

We aim to provide a critical perspective by examining the strength of the evidence, the reproducibility of mechanisms, and the primary uncertainties related to the compounds analyzed. The classification of the compounds into three evidence tiers is based on the number of independent studies conducted and the range of model systems used. Tier 1 (strongest evidence; ≥3 independent studies) comprises quercetin and resveratrol. Tier 2 (moderate evidence; 2 studies) includes nobiletin and oxymatrine. Tier 3 (emerging evidence; single study) encompasses the remaining compounds, including urolithin A, melatonin, genistein, curcumin, fisetin, S-allylcysteine, *Panax japonicus* saponins, homoplantaginin, honokiol, sesamin metabolites, EGCG, ginseng oligopeptides, and astragalus polysaccharides.

In relation to pathway convergence, two mechanisms show significant support across multiple studies. The PINK1/Parkin-dependent mitophagy pathway is consistently influenced by nobiletin, melatonin, curcumin, and starfish extract, while the SIRT1/AMPK/NAD^+^ antioxidative pathway is activated by resveratrol, ginseng oligopeptides, astragalus polysaccharides, oxymatrine, and quercetin, highlighting these as legitimate and pharmacologically accessible nodes.

In addition, several findings emphasize the complexities that are context-dependent. For example, quercetin functions through different mediators—SIRT1, NF-κB, Nrf2 or miR-34a—according to the cell type [53,54,55,56]. Similarly, mitochondrial dynamics play a dual role; while hyperfusion is the norm in most senescent cells, excessive fragmentation can also trigger senescence in particular contexts (such as COPD lung tissue and OPA1-deficient MSCs) [22,23]. Moreover, urolithin A engages DRP1-dependent fission to promote mitophagy, indicating that effective intervention may require context-specific adjustments rather than a uniform restoration of a single morphological state [64]. The pleiotropic characteristics of various compounds, especially quercetin, nobiletin, and urolithin A, emphasize the difficulty in attributing singular mechanisms of action and highlight the necessity for systematic multi-target profiling in forthcoming research.

Ultimately, several significant gaps hinder translational confidence: (i) the total lack of head-to-head comparative efficacy studies; (ii) a strong dependence on acute chemically induced senescence models instead of naturally aged tissues; (iii) an almost complete absence of human data; and (iv) the lack of studies that combine these natural compounds with optimized mitochondrial delivery systems in senescence-specific models. Tackling these deficiencies is essential for future research.

## 7. The Challenge of Precise Mitochondrial Targeting and Emerging Nanoparticle Solutions

### 7.1. Comparative Analysis of Mitochondria-Targeted Delivery Platforms

To support logical platform selection and to underscore translational readiness, the four nanoparticle platforms discussed below are analyzed with respect to their advantages, limitations, toxicity profiles, and status in translation (Table 2). TPP-based systems represent some of the most thoroughly studied platforms, utilizing ΔΨm-dependent accumulation [67]. Nonetheless, they are hindered by non-specific mitochondrial aggregation, potential interference with ΔΨm, and a constrained capacity for delivering large biomolecules. Mitochondria-penetrating peptides (MPPs), including SS-31 (elamipretide), have the ability to enter mitochondria without dependence on membrane potential [68,69]. These systems provide low toxicity and advantageous targeting; however, the specificity is still not optimal in the absence of senescent-selective targeting. Liposome-based systems, like MITO-Porter, facilitate delivery through membrane fusion, thereby circumventing endosomal pathways [70], and while effective for liver delivery, they encounter scalability issues in the context of clinical translation. Polymer-based nanoparticles provide adjustable release profiles and functionalization options, for instance; neurotransmitter-derived lipid platform shows consistent BBB penetration; however, they are still in the preliminary preclinical phase, raising concerns regarding their biocompatibility [71]. Although biomimetic carriers provide immune evasion and enhanced targeting capabilities, they are constrained by the complexities of manufacturing, high costs, and regulatory uncertainties. All four platforms have demonstrated efficacy in preclinical in vivo models, including those for Alzheimer’s disease, drug-induced liver injury, ischemic stroke, and myocardial ischemia–reperfusion injury [67,68,69,70].

However, no delivery platform has yet been combined with a natural senotherapeutic compound and specifically tested for targeting senescent cells in vivo. Therefore, the selection of a platform should be based on the characteristics of the compound. The integration of mitochondria-targeted delivery systems with natural senotherapeutics remains a hypothetical but promising future direction, rather than an established approach supported by current experimental evidence.

### 7.2. Senescence-Activated Prodrugs and Delivery Systems

Existing literature provides ample evidence supporting the use of SA-β-gal-activatable prodrugs and galactose-conjugated nanocarriers for the precise targeting of senescent cells. Small-molecule prodrugs are recognized as the most widely studied method. The β-galactosidase-targeted prodrug SSK1 has proven effective in eliminating senescent cells in both mouse and human systems across a range of tissues and cell types, which results in reduced inflammation and the restoration of physical capabilities in aged mice [72,73]. Furthermore, these SSK1-loaded nanoparticles have been effective in reducing the accumulation of senescent cells, mitigating amyloid-beta-related pathology, and improving cognitive abilities in models of Alzheimer’s disease in mice [71]. Likewise, duocarmycin prodrugs that have been modified with galactose are being utilized as senolytics, and an antibody-drug conjugate that focuses on B2M has been found to successfully clear senescent cells by specifically releasing duocarmycin payloads within those cells [74].

Nanoparticle-based methodologies have advanced this paradigm. Galactose-functionalized micelles have been engineered for targeted delivery of the senolytic Bcl2-inhibitor Navitoclax to senescent cells, significantly reducing off-target toxicity and enhancing the senolytic index [75]. Similarly, Nav-Gal—a galactose-conjugated derivative of Navitoclax—releases the active drug preferentially after galactose hydrolysis in senescent cells, where SA-β-gal activity is heightened [76]. Additionally, SA-β-gal-activatable prodrugs have emerged as a preclinical senolytic strategy that conceals drugs with galactose or similar caging groups, allowing selective unmasking in cells with high lysosomal SA-β-gal activity [72,77]. For instance, the prodrug TSPD enables fluorescent tracking and specific release of the parent drug gemcitabine in β-gal-enriched cells following SA-β-gal activation [78]. These studies confirm SA-β-gal as a promising trigger for activating senescence-specific drugs.

It is crucial to highlight that the choice concerning senolytic therapy within a clinical environment should be determined by a qualified clinician, given that the information presented here originates from preclinical models and has not yet confirmed efficacy or safety in humans.

### 7.3. Translational Barriers and Clinical Perspectives

Despite the compelling preclinical evidence presented above, translating mitochondria-targeted natural senotherapeutics from bench to bedside faces a series of interconnected barriers that must be systematically addressed. The journey begins with pharmacokinetic limitations, as most natural compounds—exemplified by oxymatrine and resveratrol—suffer from poor oral bioavailability, extensive first-pass metabolism, and rapid clearance, severely restricting the concentrations that reach target tissues and mitochondria [47,51,52]. Compounding this issue, dose feasibility is complicated by the high doses required to achieve therapeutic mitochondrial concentrations in vivo, while the hormetic nature of many natural compounds—where beneficial effects may reverse at higher doses—demands careful dose optimization. Even if adequate exposure is achieved, long-term safety remains largely unknown, particularly in elderly populations with polypharmacy and age-related organ dysfunction, as most studies evaluate short-term administration in acute models rather than chronic regimens relevant to aging.

Further complicating clinical development, standardization of botanical preparations poses substantial challenges due to inherent batch-to-batch variability in composition, necessitating rigorous quality control and validated analytical methods to ensure reproducibility. Inter-individual variability adds another layer of complexity, arising from genetic polymorphisms in drug-metabolizing enzymes, gut microbiome differences (critically influencing compounds like urolithin A) [44], and age-related physiological changes, all of which complicate universal dosing strategies. Finally, and perhaps most critically, no clinical trial has yet evaluated a mitochondria-targeted natural compound with senescence or mitochondrial endpoints, nor integrated a natural senotherapeutic with a delivery platform—a gap that must be prioritized for the field to advance.

## 8. Conclusions

Mitochondrial dysfunction is a major factor in cellular senescence, involving inadequate removal of damaged mitochondria, increased oxidative stress, altered dynamics, and inflammation from mitochondrial DNA. Evidence indicates that bioactive compounds can alleviate these issues in preclinical models, but their therapeutic potential is constrained by challenges in effective delivery to mitochondria in living organisms.

To translate these findings into clinical practice, several key research priorities must be addressed. First, standardized senescence models are needed, as most studies use chemically induced models that may not reflect chronic aging; validation with naturally aged tissues and patient-derived cells is essential. Second, multi-omics approaches at single-cell resolution will help identify molecular signatures contributing to mitochondrial dysfunction in senescence. Third, biomarker-guided patient stratification using markers like mtDNA copy number and SASP factors is crucial for identifying individuals likely to benefit from senotherapies. Fourth, optimizing mitochondria-targeted delivery systems should focus on senescence-specific “dual-lock” strategies. Finally, well-structured clinical trials with appropriate endpoints, long-term follow-up, and safety monitoring in elderly populations are necessary.

In order to expedite the process of translation, we propose the following suggestions for essential stakeholders. Researchers ought to focus on validation within naturally aged models, perform direct comparisons of compounds, and adopt multi-omics methodologies. Clinicians are encouraged to participate in the design of trials, aid in the development of biomarkers, and oversee the long-term safety and interactions between herbs and drugs in older populations. Industry experts should allocate resources towards mitochondria-targeted delivery systems, maintain stringent quality control and standardization of botanical products, and investigate combination strategies. Policymakers and funding organizations must establish regulatory frameworks for natural products, allocate specific funding for translational research, and promote standardization efforts and collaborative networks.

The assumption that natural compounds are inherently safe due to their dietary history requires critical examination. Dietary exposure typically involves low concentrations and minimal absorption, while therapeutic applications—especially with mitochondrial delivery systems—demand higher concentrations. At these pharmacologically active levels, the safety profile may change, and potential toxicity, off-target effects, and drug interactions should not be trivialized. Therefore, comprehensive toxicological evaluations at therapeutic doses are necessary before clinical use.

The available tools are easily accessible. The primary aim is to merge the investigation of mitochondrial delivery with senescence biology within a unified experimental framework, intending to create a new class of natural senotherapeutics that specifically target mitochondria—thereby significantly extending healthspan. Crucially, the experimental demonstration of integrating mitochondria-targeted delivery systems with natural senotherapeutics has yet to occur, rendering this combination a theoretical notion that necessitates thorough exploration in forthcoming research. The field is currently in an exciting yet nascent phase, necessitating systematic, rigorous, and cautious advancement to convert these mechanistic insights into significant clinical applications for aging and age-related conditions.

## Figures and Tables

**Table 1:** Summary of natural compounds targeting mitochondrial mechanisms in cellular senescence.

Compound	Target Pathway	Experimental Model	Principal Findings	Ref.
Nobiletin	PINK1/Parkin mitophagy; AMPK/SIRT1	In vitro (chicken granulosa cells)	Activates AMPK/SIRT1 upstream of PINK1/Parkin; reduces D-gal-induced senescence	[41]
Melatonin	PINK1/Parkin mitophagy	In vivo (mouse kidney)	Prevents atrazine-induced renal senescence; Parkin-dependent	[40]
Curcumin	PINK1/Parkin mitophagy	In vitro (rat BM-MSCs)	Upregulates PINK1, Parkin, LC3-II/I; reduces p62; mitophagy-dependent	[42]
Starfish extracts	PINK1/Parkin mitophagy	In vitro (human dermal fibroblasts)	Reduces SA-β-gal, p21, MMP-1, IL-6, IL-8; PINK1-dependent	[43]
Urolithin A	Parkin + BNIP3 mitophagy	In vitro (auditory cells)	Restores ATP, mtDNA, ΔΨm; requires both Parkin and BNIP3	[44]
Genistein	ERRα-mediated mitophagy + biogenesis	In vitro (rat BM-MSCs from ovariectomized rats)	ERRα-dependent mitophagy and biogenesis; reduces ROS and senescence	[45]
Resveratrol	SIRT1/FOXO3; SIRT1-Nrf2-FOXO3	In vitro (skin, ovarian models)	Increases antioxidant enzymes; reduces oxidative stress, inflammation, ferroptosis; inhibits MAPK	[46,47,48]
Ginseng oligopeptides	NAD^+^/SIRT1/PGC-1α	In vitro (fibroblasts)	Enhances mitochondrial function and biogenesis; reduces p16, p21; boosts telomerase	[49]
Astragalus polysaccharides	SIRT1-p53	In vitro (rat aortic endothelium)	Restores ΔΨm; enhances antioxidant capacity	[50]
Oxymatrine	AMPK; Nrf2-SIRT3	In vitro (HT22 neurons, cardiac cells); in vivo (mouse)	Reduces ROS; reactivates AMPK; improves mitochondrial function in brain and cardiac senescence	[51,52]
Quercetin	SIRT1; NF-κB; Nrf2; miR-34a/SIRT1	In vitro (multiple cell types: MSCs, fibroblasts, periodontal cells, adipocytes)	Cell-type dependent: SIRT1 activation, NF-κB suppression, Nrf2 activation, or miR-34a/SIRT1 axis; reduces ROS, SA-β-gal, inflammatory cytokines	[53,54,55,56]
Fisetin	Nrf2/HO-1	In vitro (follicular granulosa cells)	Activates Nrf2/HO-1; restores mitochondrial function; reduces p53, p21	[57]
Sesamin metabolites	Direct ROS scavenging	In vitro (fibroblast)	Lowers mtROS; improves ROS/ATP ratio; reduces SASP and DNA damage	[58]
EGCG	Secretome	In vitro (endothelial cells)	Inhibits SA-β-gal; reverses p21, p16, p15; reduces IL-6, IL-8	[59]
S-allylcysteine	MFN2/OPA1 (fusion)	In vivo (naturally aged mice); In vivo (*C. elegans*)	Increases OPA1, SIRT1, PGC-1α; preserves mitochondrial morphology; reduces oxidative damage and senescence markers	[62]
*Panax japonicus* saponins	MFN2/OPA1 (fusion); DRP1 (fission)	In vivo (aged rat hippocampus); In vitro (SH-SY5Y cells)	Increases MFN2, OPA1; reduces DRP1; improves mitochondrial structure; reduces D-gal-induced aging	[63]
Homoplantaginin	mtDNA-cGAS-STING (mtDNA leakage)	In vitro (HUVECs); In vivo (diabetic mouse)	Inhibits DRP1-mediated fission and VDAC1 aggregation; prevents mtDNA release; suppresses cGAS-STING; reduces SA-β-gal and SASP	[65]
Honokiol	mtDNA-cGAS-STING (oxidative damage)	In vivo (silicosis mouse model)	Activates SIRT3; enhances SOD2; reduces mtDNA oxidative damage; inhibits STING/NF-κB; reduces senescence and fibrosis	[66]

**Table 2:** Comparison of mitochondria-targeted delivery platforms.

Platform	Advantages	Limitations	Toxicity	Translational Status
FGFR1-targeting + SS-31/Humanin [68]	High brain accumulation; specificity to cholinergic neurons	Short safety window tested	No obvious toxicity	Preclinical (mouse model)
MITO-Porter (liposome) [70]	High liver accumulation; reaches hepatocytes	Short-term acute model	No toxicity reported	Preclinical (mouse model)
ROS-responsive polymer + SS-31 [69]	Blood–brain barrier bypass; mitochondrial specificity (in vitro)	Small sample size	Quite safe	Preclinical (mouse model)
TPP + cardiac-homing peptide [67]	Dual targeting; mitochondrial specificity (in cell line)	Short biodistribution window tested	No systemic toxicity tested	Preclinical (mouse model)

## Data Availability

No new data were created or analyzed in this study. Data sharing is not applicable to this article.

## Abbreviations

The following abbreviations are used in this manuscript:

5-FU	5-Fluorouracil
8-OHdG	8-Hydroxy-2′-deoxyguanosine
AIF	Apoptosis-Inducing Factor
AKT	Protein Kinase B (PKB)
AMPK	AMP-activated protein kinase
ATM	Ataxia Telangiectasia Mutated
ATP	Adenosine Triphosphate
BBB	Blood–Brain Barrier
BM-MSC	Bone Marrow-Derived Mesenchymal Stem Cell
BNIP3	BCL2 Interacting Protein 3
cGAS	Cyclic GMP-AMP Synthase
CoA	Coenzyme A
COPD	Chronic Obstructive Pulmonary Disease
CoQ10	Coenzyme Q10 (ubiquinone)
COX-2	Cyclooxygenase-2
CXCL1	C-X-C Motif Chemokine Ligand 1
CXCL8	C-X-C Motif Chemokine Ligand 8
DAMP	Danger-Associated Molecular Pattern
D-gal	D-Galactose
DMOG	Dimethyloxalylglycine
DNA	Deoxyribonucleic Acid
DRP1	Dynamin-Related Protein 1
EGCG	Epigallocatechin Gallate
ERRα	Estrogen-Related Receptor Alpha
FAO	Fatty Acid Oxidation
FGFR1	Fibroblast Growth Factor Receptor 1
FIS1	Fission 1
FOXO3	Forkhead Box O3
GDC0068	Ipatasertib (pan-AKT inhibitor)
GLB1	Galactosidase Beta 1
GLS1	Glutaminase 1
GM-CSF	Granulocyte-Macrophage Colony-Stimulating Factor
GSH-Px	Glutathione Peroxidase
H_2_O_2_	Hydrogen Peroxide
HELLS	Helicase, Lymphoid Specific
HIF-1α	Hypoxia-Inducible Factor 1-Alpha
HNSS	Hybrid peptide (SS-31 + Humanin)
HO-1	Heme Oxygenase-1
Humanin	Mitochondrial-derived peptide (not an acronym)
HUVECs	Human Umbilical Vein Endothelial Cells
ICAM-1	Intercellular Adhesion Molecule 1
IDH2	Isocitrate Dehydrogenase 2
IFN	Interferon
IL-1β	Interleukin-1 Beta
IL-6	Interleukin-6
IL-8	Interleukin-8
LC3	Microtubule-Associated Protein 1A/1B-Light Chain 3
MAPK	Mitogen-Activated Protein Kinase
MDA	Malondialdehyde
MFF	Mitochondrial Fission Factor
MFN1	Mitofusin 1
MFN2	Mitofusin 2
MIEF1	Mitochondrial Elongation Factor 1
MID49/MID51	Mitochondrial Dynamics Proteins of 49/51 kDa
miR-34b/c	microRNA-34b/c
MITO-Porter	Liposome-based mitochondrial delivery system (proper name)
MitoQ	Mitoquinone (TPP-conjugated ubiquinone)
MitoTEMPO	Mitochondria-targeted TEMPO
MMP-1	Matrix Metalloproteinase-1
MMP-3	Matrix Metalloproteinase-3
mPTP	Mitochondrial Permeability Transition Pore
mtDNA	Mitochondrial DNA
mtROS	Mitochondrial Reactive Oxygen Species
NAD^+^	Nicotinamide Adenine Dinucleotide (oxidised)
NADH	Nicotinamide Adenine Dinucleotide (reduced)
NF-κB	Nuclear Factor Kappa-Light-Chain-Enhancer of Activated B Cells
NLRP3	NOD-, LRR- and Pyrin Domain-Containing Protein 3
NOX1	NADPH Oxidase 1
Nrf2	Nuclear Factor Erythroid 2-Related Factor 2
OPA1	Optic Atrophy 1
OXPHOS	Oxidative Phosphorylation
p15	Cyclin-Dependent Kinase Inhibitor 2B (CDKN2B, p15^INK4b^)
p16	Cyclin-Dependent Kinase Inhibitor 2A (CDKN2A, p16^INK4a^)
p21	Cyclin-Dependent Kinase Inhibitor 1A (CDKN1A, p21^CIP1^)
p53	Tumor Protein p53 (TP53)
p62	Sequestosome-1 (SQSTM1)
Parkin	Parkinson Juvenile Disease Protein 2 (PRKN)
PD1	Programmed Cell Death Protein 1 (CD279)
PDK4	Pyruvate Dehydrogenase Kinase 4
PGC-1α	Peroxisome Proliferator-Activated Receptor Gamma Coactivator 1-Alpha
PINK1	PTEN-Induced Putative Kinase 1
RA	Rheumatoid Arthritis
ROS	Reactive Oxygen Species
SA-β-gal	Senescence-Associated Beta-Galactosidase
SASP	Senescence-Associated Secretory Phenotype
SC1, EC1-2	Sesamin Metabolites
SH-SY5Y	Human neuroblastoma cell line
shRNA	Short Hairpin RNA
siRNA	Small Interfering RNA
SIRT1	Sirtuin 1
SIRT3	Sirtuin 3
SIRT4	Sirtuin 4
SOD	Superoxide Dismutase
SOD2	Superoxide Dismutase 2 (manganese-dependent)
SPJ	*Panax japonicus* Saponins
SS-31	Szeto-Schiller Peptide 31 (elamipretide)
STING	Stimulator of Interferon Genes
TLR9	Toll-Like Receptor 9
TNF-α	Tumour Necrosis Factor Alpha
TPP	Triphenylphosphonium
UA	Urolithin A
VCAM-1	Vascular Cell Adhesion Molecule 1
VDAC	Voltage-Dependent Anion Channel
VDAC1	Voltage-Dependent Anion-Selective Channel 1
β-gal	Beta-Galactosidase
γH2A.X	Phosphorylated H2A Histone Family Member X
